# Immune activation by a multigene family of lectins with variable tandem repeats in oriental river prawn (*Macrobrachium nipponense*)

**DOI:** 10.1098/rsob.200141

**Published:** 2020-09-16

**Authors:** Ying Huang, Xin Huang, Xuming Zhou, Jialin Wang, Ruidong Zhang, Futong Ma, Kaiqiang Wang, Zhuoxing Zhang, Xiaoling Dai, Xueying Cao, Chao Zhang, Keke Han, Qian Ren

**Affiliations:** 1 College of Marine Science and Engineering, Nanjing Normal University, 1 Wenyuan Road, Nanjing, Jiangsu 210023, People's Republic of China; 2 College of Oceanography, Hohai University, 1 Xikang Road, Nanjing, Jiangsu 210098, People's Republic of China; 3 Co-Innovation Center for Marine Bio-Industry Technology of Jiangsu Province, Lianyungang, Jiangsu 222005, People's Republic of China; 4 Hubei Key Laboratory of Genetic Regulation and Integrative Biology, School of Life Sciences, Central China Normal University, Wuhan 430079, People's Republic of China; 5 Key Laboratory of Animal Ecology and Conservation Biology, Institute of Zoology, Chinese Academy of Sciences, Beijing, People's Republic of China

**Keywords:** tandem repeats, lectin, slip mispairing, alternative splicing, functional diversity, innate immunity

## Abstract

Genomic regions with repeated sequences are unstable and prone to rapid DNA diversification. However, the role of tandem repeats within the coding region is not fully characterized. Here, we have identified a new hypervariable C-type lectin gene family with different numbers of tandem repeats (Rlecs; R means repeat) in oriental river prawn (*Macrobrachium nipponense*)*.* Two types of repeat units (33 or 30 bp) are identified in the second exon, and the number of repeat units vary from 1 to 9. Rlecs can be classified into 15 types through phylogenetic analysis. The amino acid sequences in the same type of Rlec are highly conservative outside the repeat regions. The main differences among the Rlec types are evident in exon 5. A variable number of tandem repeats in Rlecs may be produced by slip mispairing during gene replication. Alternative splicing contributes to the multiplicity of forms in this lectin gene family, and different types of Rlecs vary in terms of tissue distribution, expression quantity and response to bacterial challenge. These variations suggest that Rlecs have functional diversity. The results of experiments on sugar binding, microbial inhibition and clearance, regulation of antimicrobial peptide gene expression and prophenoloxidase activation indicate that the function of Rlecs with the motif of YRSKDD in innate immunity is enhanced when the number of tandem repeats increases. Our results suggest that Rlecs undergo gene expansion through gene duplication and alternative splicing, which ultimately leads to functional diversity.

## Key points

1. A hypervariable C-type lectin gene family was found in prawn.
2. Diversity of Rlecs can be produced by slip mispairing and alternative splicing.
3. Variation in the number of tandem repeats influenced the immune function of Rlecs.

## 1. Introduction

Repetitive DNA sequences are widespread and abundant in genomic DNAs because almost half of the human genome consists of repeats [1,2]. A subset of repeating DNA are DNA fragments consisting of tandem repeats with short sequence units (e.g. CAG) that are adjacent to each other. The terms ‘microsatellites’ and ‘minisatellites’ are often used to represent tandem repeats with short (less than or equal to 9 bp) and long (greater than 9 bp) repeat units, respectively. Tandem repeats can be mutation hotspots due to their repetitive features. Slippage during DNA replication or recombination events results in alleles with different numbers of repeat units, which are referred to as ‘copy numbers’. Tandem repeats have higher mutation rates, which are 10–10 000 times the average rate, compared with other genomic loci [3]. Most tandem repeats lose direct biological functions due to instability and the lacking genetic information. Such tandem repeats are referred to as ‘junk’ DNA [4,5]. However, tandem repeats are useful as genetic markers in genotyping and forensic science and offer additional advantages for genome-wide linkage studies [6]. Furthermore, repeats are present in the functional (coding and regulatory) regions of genomes [7] and can alter the function and/or expression of genes to enable organisms to adapt quickly to new environments [8].

Repetitive sequences are important promoters of biological genomic DNA evolution, but the origin and evolutionary mechanism of tandem repeats have been controversial [9]. An early explanation for this variation is that DNA slip mismatch occurs during replication, and the DNA is then repaired and recombined to produce a repetitive sequence [10]. However, no study has fully explained the diversity of repeat sequence types within the same genome, and between the coding and the non-coding regions. Host defence proteins are important in combating microbial infections. Few excess tandem repeat variations have been observed in human defence proteins, but tandem repeat polymorphisms may arise in invertebrate defence proteins, which have a large population size [11]. Thus, the excessive number of tandem repeat polymorphisms in invertebrate defence proteins needs further investigation.

Host immunity is a continuous game between host and pathogen. Pathogens can invade the host quickly and efficiently, and the immune system is responsible for protecting the host from pathogens. This long-term coevolution between hosts and pathogens undergoes a process in which the mutation rate of long-generation hosts is low, whereas that of short-generation microorganisms is high [12]. This evolutionary mechanism enables hosts to protect themselves effectively against pathogens that show evolutionary variations. The best option for the host is to find ways to generate random or near-random diversification and expand immune receptors.

Lectins are pattern recognition receptors (PRRs) that are actively involved in various life processes, including protein trafficking, cell adhesion, phagocytosis, cell signalling, complement activation and non-self recognition [13]. Lectins have carbohydrate recognition domains (CRDs), which bind to sugars. Different CRDs have various symbolic structures with unique amino acid motifs that can recognize specific types of sugars [14]. Among the lectins, C-type lectins (CTLs) are the most abundant and widely studied [15]. The main characteristic of CTLs is the presence of at least one CRD containing a highly conserved Ca^2+^ binding site 2 with a QPD (Gln–Pro–Asp) or EPN (Glu–Pro–Asn) motif, which is specific for galactose or mannose binding, respectively [16]. CTLs, which exist in almost all metazoans, are highly conserved in vertebrates and considerably diverse in invertebrates [15]. A variety of CTLs is present in one species and varies widely in amino acid sequences. However, these CTLs do not belong to a single family. A growing number of crustacean CTLs have been identified as PRRs or effectors participating in a series of immune defence responses [15]. The presence of tandem repeat polymorphisms in a single family of CTLs, as a kind of host defence protein, has not been studied.

Here, we have identified a hypervariable CTL gene family with tandem repeat polymorphisms (Rlecs; R means repeat) in oriental river prawn (*Macrobrachium nipponense*)*.* A common feature of this lectin gene family is that all Rlecs have variable numbers of tandem repeats in the coding regions outside the CRDs. The number of repeat units varies from 1 to 9. Moreover, we have characterized the arrangement patterns of the repeat units in this lectin gene family. Rlecs with a motif of six different amino acids can be classified into 15 types through phylogenetic analysis. We have also determined the tissue distributions and expression patterns of eight types of Rlecs under bacterial challenge. Finally, a functional study on Rlecs–YRSKDD with variable numbers of tandem repeats (1, 2, 7 or 9) in innate immunity is conducted.

## 2. Results

### 2.1. Characterization and sequence analysis of Rlecs

We found a lectin gene containing tandem repeats by analysing the transcriptome data of *M. nipponense*. More than one band was amplified using a pair of gene-specific primers (data not shown). After sequencing many clones, we found that these lectin genes belong to a multiple-gene family. These lectin genes had tandem repeats and were named as *Rlecs*. A common feature of these lectin genes was their tandem repeat region. Their tandem repeat unit can be either 33 or 30 bp long, and the number of tandem repeat unit varied from 1 to 9 (electronic supplementary material, table S1).

We selected a *Rlec* with YRSKDD motif (*Rlec*–YRSKDD) as an example to describe the characteristics of *Rlec* gene sequences in detail. The full-length cDNA of *Rlec*–YRSKDD that contained nine tandem repeats was 1408 bp, including an 894 bp open reading frame that encoded a 297-amino acid protein, a 47 bp 5′ untranslated region (UTR) and a 467 bp 3′ UTR with a canonical polyadenylation signal site (AATAAA) (electronic supplementary material, figure S1a). The repeat region of *Rlec*–YRSKDD had 279 bp, and the array of the nine tandem repeats was 33–30–30–30–30–30–33–30–33. The multiple alignments of the nine tandem repeats indicated that they were highly conserved (electronic supplementary material, figure S1b). Rlec–YRSKDD protein had a putative signal peptide containing 30 amino acid residues, 2 internal repeats (amino acids 59–96 and 109–148) and one CRD (amino acids 159–283). EPN (Glu^248^–Pro^249^–Asn^250^) was found in the CRD of Rlec–YRSKDD. In addition, the theoretical isoelectric point and the molecular weight of Rlec–YRSKDD were 5.10 and 32.24 kDa, respectively.

### 2.2. Phylogeny and genomic sequence analysis

**Figure 1.**
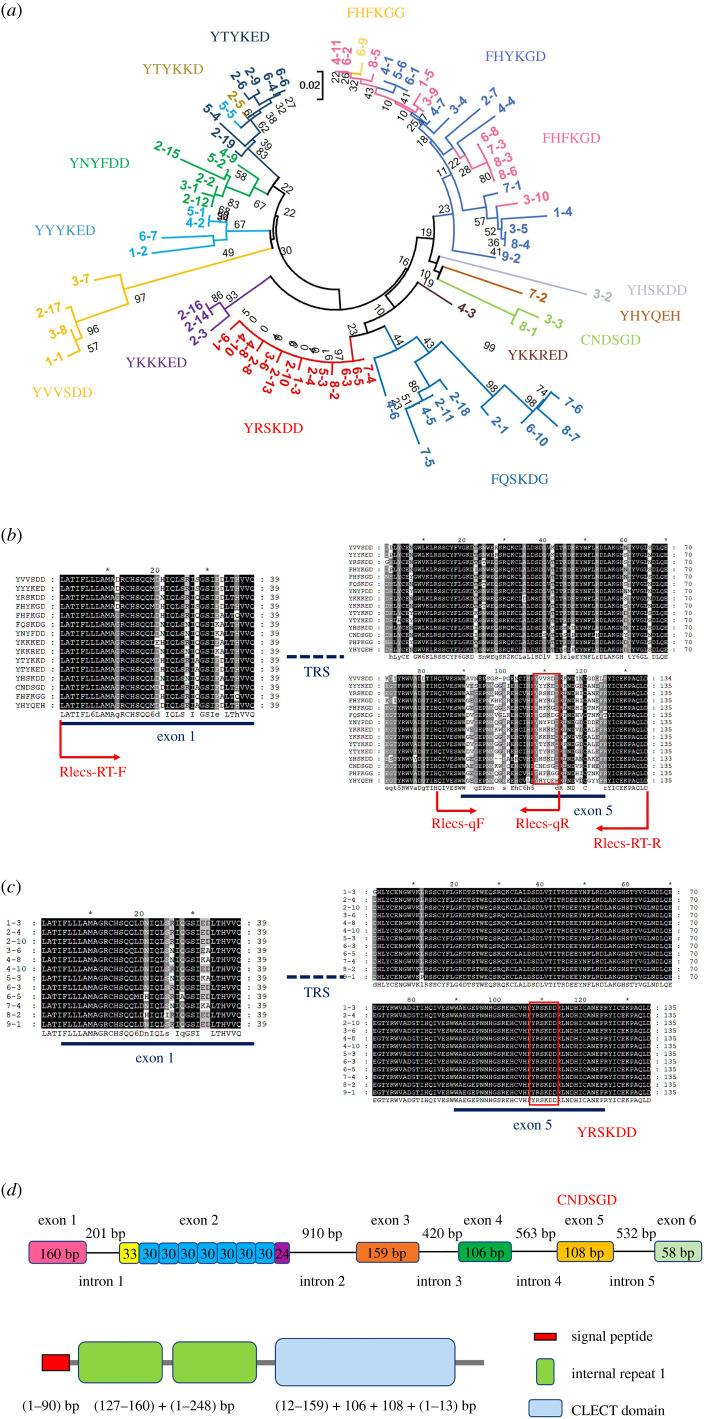
Sequence analysis and genome structure of *Rlecs* from *M. nipponse*. (*a*) Phylogenetic tree constructed using the amino acid sequences of 76 Rlecs. Bootstrap trials were replicated 1000 times to derive the confidence value. (*b*,*c*) Multiple alignments of (*b*) the 15 different types of Rlecs and (*c*) Rlecs–YRSKDD by using the amino acid sequences without repeat units. Absolutely consistent sites and highly consistent sites are coloured in dark and grey backgrounds, respectively. (*d*) Genomic structure and domain architecture of *Rlec*–CNDSGD with eight tandem repeats. The coding region of *Rlec*–CNDSGD-8 contains six exons interrupted by five introns. The repeat region is located in exon 2.

A number of clones were selected and sequenced to study the diversity of these Rlecs. The same sequences were removed, and a total of 76 Rlecs (GenBank number in electronic supplementary material, table S1) were used to construct the phylogenetic tree by using the neighbour-joining method. All Rlecs can be clustered into 15 types. Each type of Rlecs had a unique motif with six amino acids. The Rlecs were classified into CNDSGD-, FHFKGG-, FHFKGD-, FHYKGD-, FQSKDG-, YHSKDD-, YHYQEH-, YKKKED-, YKKRED-, YNYFDD-, YRSKDD-, YTYKED-, YTYKKD-, YVVSDD- and YYYKED-type Rlecs (figure 1*a*).

The multiple alignments of the 15 different types of Rlecs by using amino acid sequences before and after the repeat region indicated that the main sequential variance came from the fifth exon (figure 1*b*). The YRSKDD-type was used as an example to study the differences of the same type of Rlecs. Multiple alignments showed that the amino acid sequences of the same type of Rlecs were highly conserved (figure 1*c*). The genomic sequences of *Rlec* isoforms varied, but their genome structures were probably similar to each other. All *Rlec* isoforms contained six exons interrupted by five introns. The repeat region was located in the second exon. The length and nucleotide sequences of introns in the *Rlec* isoforms also varied. The lengths of the first, third and sixth exons of the *Rlec* isoforms were not changed and were consistent with each isoform. Here, the *Rlec*–CNDSGD was used as an example. The DNA of *Rlec*–CNDSGD contained six exons (i.e. 160, 267, 159, 106, 108 and 58 bp) interrupted by five introns (i.e. 201, 910, 420, 563 and 532 bp). The eight repeat units in the repeat region (exon 2) were 33, 30, 30, 30, 30, 30, 30 and 30 bp. The Rlec–CNDSGD protein had one signal peptide, two internal repeats and one CRD. The exons corresponding to the different domains of Rlec–CNDSGD are shown in figure 1*d*.

**Figure 2.**
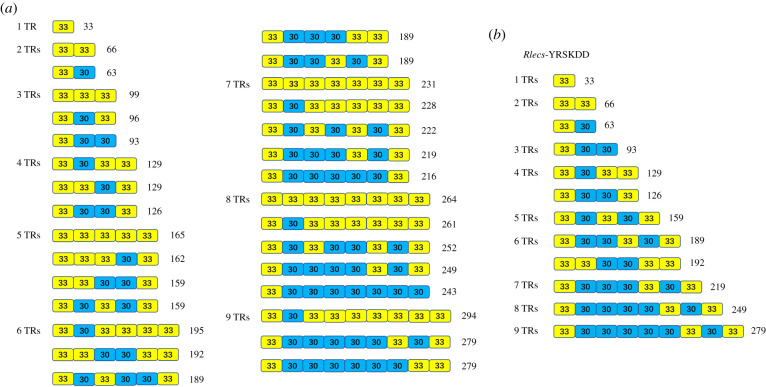
Arrangement pattern of the tandem repeats of *Rlecs* in *M. nipponense*. Arrangement pattern of tandem repeats (*a*) from different types of *Rlecs* and (*b*) of the same type of *Rlecs* (e.g. *Rlecs*–YRSKDD).

A total of 31 different arrangement patterns of the repeat units were found in 76 *Rlecs*. In detail, *Rlecs* with a tandem repeat had only one arrangement, and *Rlecs* containing two tandem repeats had two permutations. *Rlecs* containing 3, 4 and 9 tandem repeats had three permutations. *Rlecs* containing five tandem repeats had four permutations. The five different arrangement patterns of the *Rlecs* with 6, 7 and 8 tandem repeats are summarized in figure 2*a*. The repeat units of the same type of *Rlecs* (e.g. *Rlecs*–YRSKDD) also had different arrangement patterns (figure 2*b*).

### 2.3. DNA slip mismatch and alternative splicing modes of Rlecs

**Figure 3.**
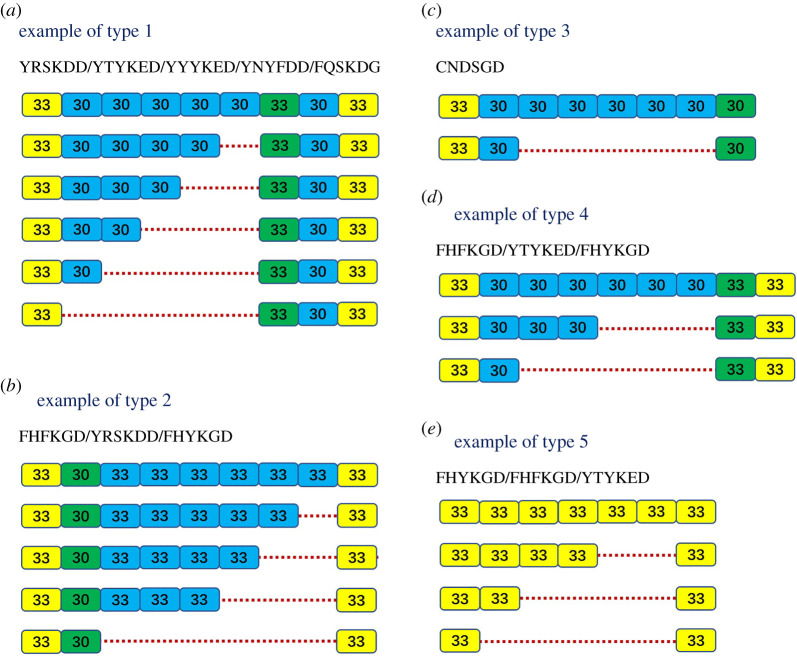
DNA slip mismatch examples of *Rlecs*. DNA sequence analysis to confirm the excision of one or more repeat units (30 or 33 bp) from the tandem repeats. The five predicted DNA slip mismatch examples in the repeat region of *Rlecs* can explain the possible mechanism of the formation of the different numbers of tandem repeats.

The expansion or contraction of nucleotides at tandem repeat regions during DNA replication may happen and is called replication slippage or slipped-strand mispairing [17]. We analysed all the arrangement patterns of the tandem repeats of 76 *Rlecs* and tried to find examples of slipped-strand mispairing to explain the possible mechanism of the formation of the different numbers of repeat units. Fortunately, five possible DNA slip mismatch examples were found. As shown in figure 3, type 1 can be described as follows. The first repeat unit and the last three repeat units were identical, and the excision of one or more 30 bp repeat units from the tandem repeat regions was found. This example can be found in the *Rlecs* containing the YRSKDD, YTYKED, YYYKED, YNYFDD or FQSKDG motif. In type 2, the first two repeat units and the last repeat unit were not changed, and one or more 33 bp repeat units were excised. This example can be found in the *Rlecs* containing the FHFKGD, YRSKDD or FHYKGD motif. In type 3, the first and the last repeat units were identical, and five repeat units with 30 bp were missing in *Rlec*–CNDSGD. Type 4 showed that the first repeat unit and the last two repeat units were the same, and several 30 bp repeat units were reduced in FHFKGD-, YTYKED- and FHYKGD-type *Rlecs*. In type 5, all repeat units were 33 bp, and several 33 bp repeat units were excised in *Rlecs* containing the FHYKGD, FHFKGD or YTYKED motif.

**Figure 4.**
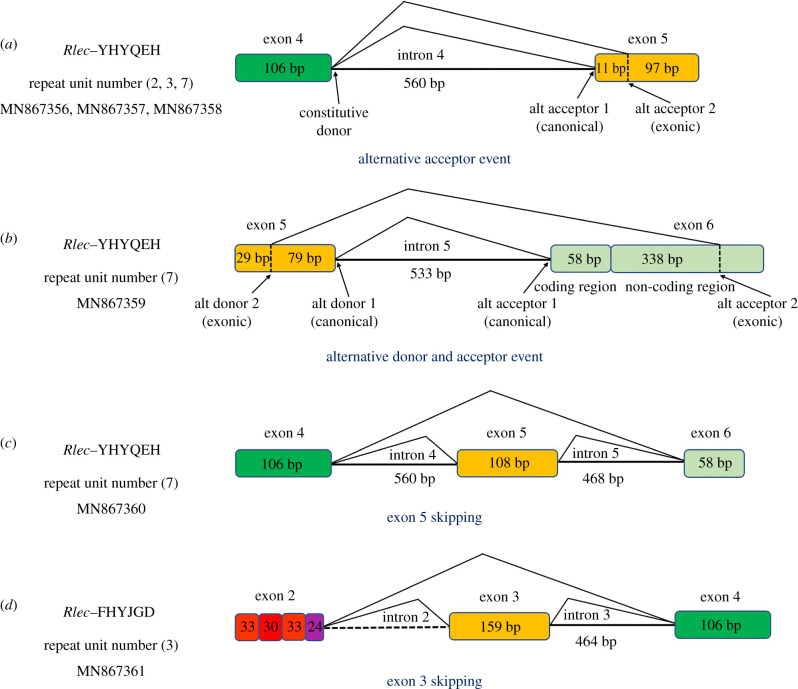
Alternative splicing modes of *Rlecs*. Sketch map of three different alternative splicing patterns (i.e. alternative acceptor event, alternative donor and acceptor event, and exon skipping) in *Rlecs*–YHYQEH and *Rlec*–FHYKGD.

Gene diversity can be produced by alternative splicing. In our research, the diversity of *Rlecs* can also be produced through alternative splicing. Three alternative modes, including alternative acceptor sites, alternative donor and acceptor sites, and exon skipping, were found in *Rlecs*. The exon–intron boundaries of the genomic sequences of the *Rlecs* were GT and AG at the 5′ and 3′ splice sites, respectively. An example of an alternative acceptor event is shown in figure 4*a*. One alternative acceptor event had two acceptor sites (canonical or exonic) and one donor site. Alternative splicing at the exonic site induced the loss of the first 11 bp in the fifth exon in one transcript, which finally produced a different gene. This splicing mode was found in *Rlecs*–YHYQEH with 2, 3 or 7 tandem repeats. Different donor sites (canonical or exonic) and different acceptor sites (canonical or exonic) were selected in another alternative splicing event (figure 4*b*). Thus, the last 79 bp in the fifth exon and the first 58 bp coding region of the sixth exon were lost in one transcript. This splicing mode was found in *Rlec*–YHYQEH with seven tandem repeats. Exon 5 skipping was found in *Rlec*–YHYQEH with seven tandem repeats (figure 4*c*), and exon 3 skipping existed in *Rlec*–FHYKGD with three tandem repeats (figure 4*d*).

### 2.4. Expression pattern analysis of the eight types of *Rlecs*

**Figure 5.**
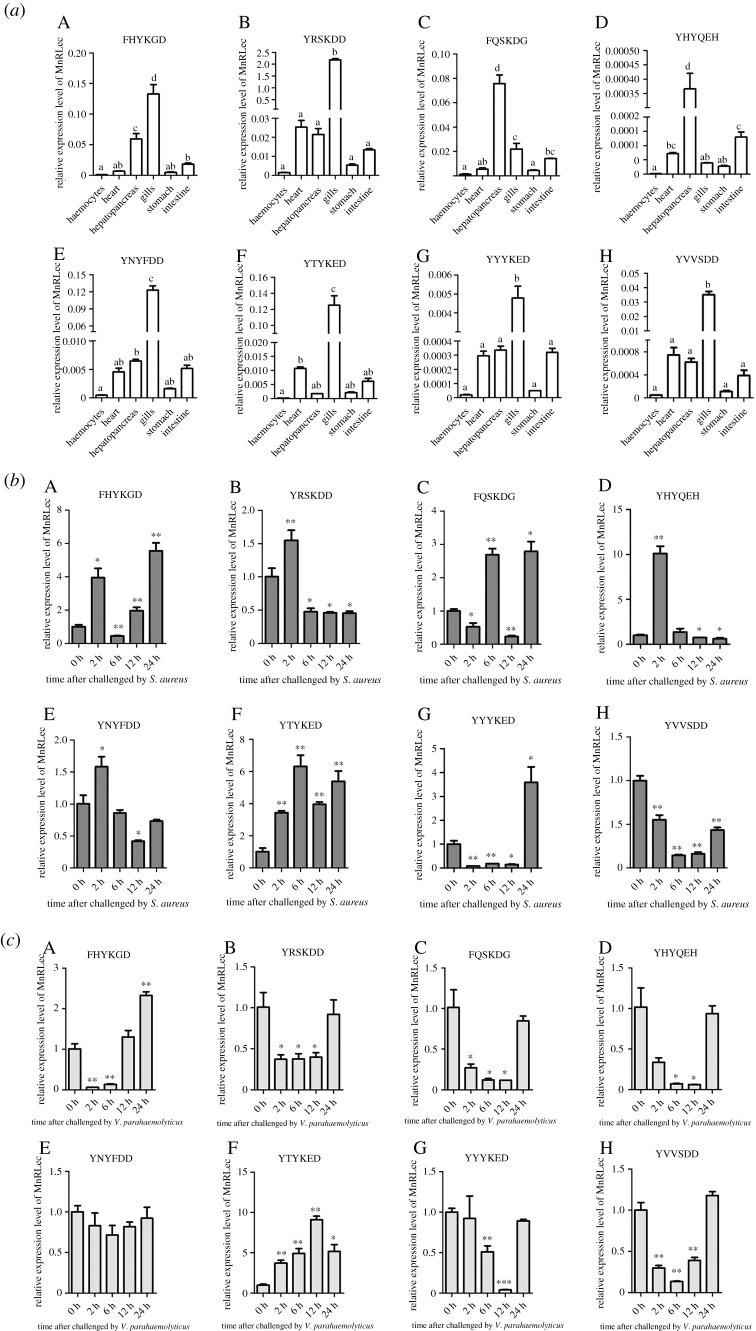
Tissue distribution and expression pattern analysis of eight types of *Rlecs*. (*a*) Expression levels of *Rlecs*–FHYKGD, *Rlecs*–YRSKDD, *Rlecs*–FQSKDG, *Rlecs*–YHYQEH, *Rlecs*–YNYFDD, *Rlecs*–YTYKED, *Rlecs*–YYYKED and *Rlecs*–YVVSDD in haemocytes, heart, hepatopancreas, gills, stomach and intestine of *M. nipponense* as determined by qRT-PCR. Different letters showed significant differences compared with other groups (*p* < 0.05). Expression patterns of the eight types of *Rlecs* in the gills of prawns challenged by *S. aureus* (*b*) and *V. parahaemolyticus* (*c*) at 0, 2, 6, 12 and 24 h as determined by qRT-PCR. β-actin was used as the reference gene for internal controls. Error bars represent the mean ± s.d. of three replicates. Asterisks indicate significant differences (*p < 0.05, **p < 0.01, ***p < 0.001) compared with control.

The 15 different types of *Rlecs* were difficult to distinguish through quantitative real-time polymerase chain reaction (qRT-PCR). Therefore, only eight types of *Rlecs* were selected to study tissue distribution and expression pattern upon different bacterial challenges. The qRT-PCR analysis results showed that all the eight types of *Rlecs* were widely expressed in haemocytes, heart, hepatopancreas, gills, stomach and intestine. However, the expression level of each type of *Rlecs* was different. *Rlecs*–FHYKGD, *Rlecs*–YNYFDD, *Rlecs*–YYYKED, *Rlecs*–YRSKDD, *Rlecs*–YTYKED and *Rlecs*–YVVSDD showed the highest expression levels in the gills, and the expression level of *Rlecs*–YRSKDD was higher than those of *Rlecs*–FHYKGD, *Rlecs*–YTYKED, *Rlecs*–YNYFDD, *Rlecs*–YVVSDD and *Rlecs*–YYYKED. *Rlecs*–FQSKDG and *Rlecs*–YHYQEH were expressed in the hepatopancreas, and the expression level of *Rlecs*–FQSKDG was higher than that of *Rlecs*–YHYQEH (figure 5*a*). The expression pattern of the eight types of *Rlecs* in the gills challenged by *Staphylococcus aureus* or *Vibrio parahaemolyticus* was further studied. The seven types of *Rlecs* (except *Rlecs*–YVVSDD) were upregulated to varying degrees at certain challenge time points upon *S. aureus* challenge (figure 5*b*). Five types of *Rlecs* were downregulated in the gills challenged with *V. parahaemolyticus*, and the expression level of *Rlecs*–YNYFDD did not change upon *V. parahaemolyticus* challenge. *Rlecs*–FHYKGD was initially reduced and then gradually increased until their highest expression level. *Rlecs*–YTYKED was upregulated at 2, 6, 12 and 24 h of *V. parahaemolyticus* challenge compared with the control (figure 5*c*).

### 2.5. Detection of antimicrobial peptide gene expression, phenoloxidase activity and bacterial clearance rates after the knockdown of *Rlecs*–YRSKDD

**Figure 6.**
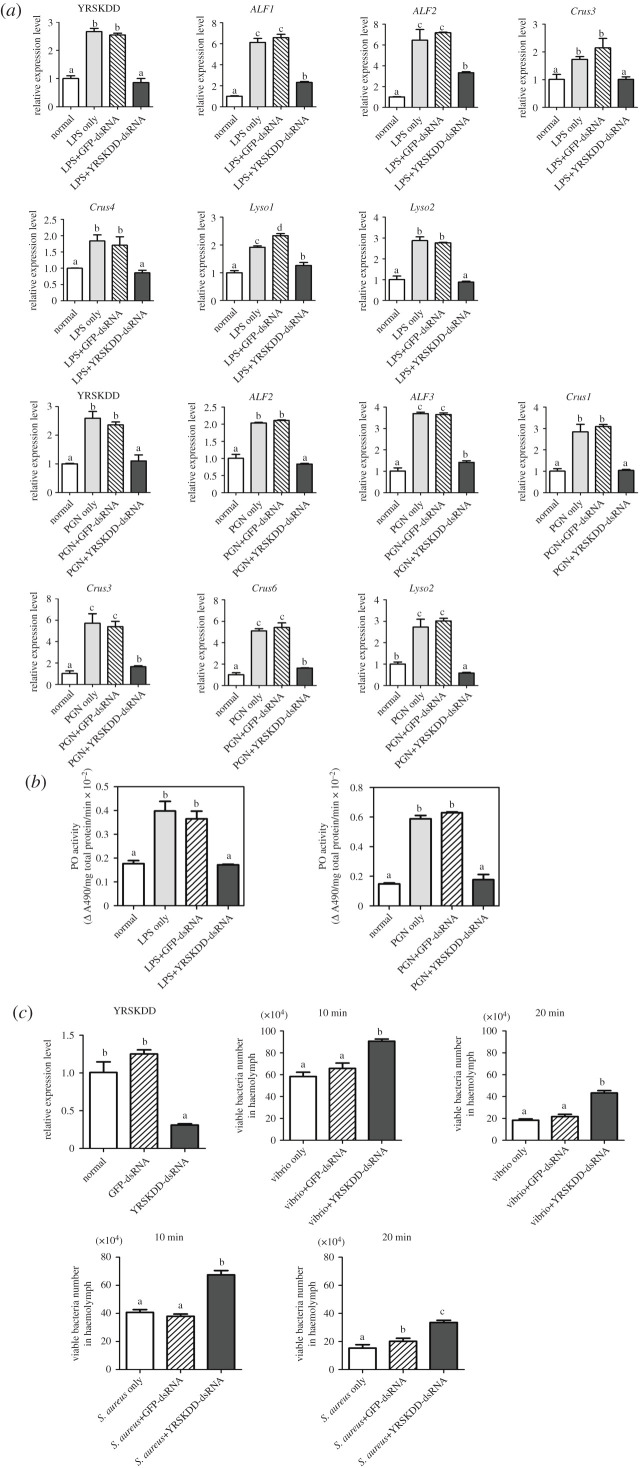
Analysis of *AMP* expression, PO activity and bacterial clearance rates after *Rlecs*–YRSKDD knockdown. (*a*) Detection of the expression of *AMPs* (*ALF1*, *ALF2*, *ALF3*, *Crus1*, *Crus3*, *Crus4*, *Crus6*, *Lyso1* and *Lyso2*) at 12 h of LPS or PGN challenge in the gills of *Rlecs*–YRSKDD knockdown prawns by using qRT-PCR. *GFP*-dsRNA plus LPS, *GFP*-dsRNA plus PGN, LPS and PGN were used as control groups. (*b*) Detection of PO activity in the gills of the seven groups (normal, LPS only, PGN only, *GFP*-dsRNA plus LPS, *GFP*-dsRNA plus PGN, *Rlecs*–YRSKDD-dsRNA plus LPS and *Rlecs*–YRSKDD-dsRNA plus PGN). The enzymatic activity of PO was measured using l-DOPA as substrate and monitored by spectrophotometry at 490 nm. (*c*) Bacterial clearance assay was performed in *Rlecs*–YRSKDD-silenced prawns. *Staphylococcus aureus* or *V. parahaemolyticus* was injected into the normal or dsRNA (*Rlecs*–YRSKDD-dsRNA or *GFP*-dsRNA)-injected prawns. Haemolymph was extracted, diluted and plated onto LB broth plates at 10 and 20 min after injection. The plates were incubated overnight at 37°C, and the number of bacterial colonies was counted. Five prawns were collected for each sample at each time point. Significant differences are marked with different letters (a, b, c and d). Data are presented as mean ± s.d. of three independent replicates. Data were analysed by ANOVA in SPSS.

On the basis of the expression level of different types of *Rlecs* in gills, the *Rlecs*–YRSKDD was selected for the RNAi experiments. As shown in figure 6*a*, the expression of *Rlecs*–YRSKDD in the gills of *Rlecs*–YRSKDD gene-derived double-stranded RNA (dsRNA)-injected prawns with 12 h of lipopolysaccharide (LPS) or peptidoglycan (PGN) challenge was downregulated compared with that of control prawns (LPS only, PGN only, green fluorescent protein [*GFP*]-dsRNA plus LPS and *GFP*-dsRNA plus PGN). The expressions of antimicrobial peptide (AMP) genes, including anti-lipopolysaccharide factor (*ALF*) 1, *ALF2*, crustin (*Crus*) 3, *Crus4*, lysozyme (*Lyso*) 1 and *Lyso2* in *Rlecs*–YRSKDD knockdown prawns, at 12 h of LPS challenge were inhibited compared with those in control prawns. The expressions of *ALF2*, *ALF3*, *Crus1*, *Crus3*, *Crus6* and *Lyso2* in the gills of *Rlec*–YRSKDD knockdown prawns after 12 h of PGN challenge decreased compared with those of the control prawns (figure 6*a*). In addition, PO activity was significantly decreased in the *Rlecs–*YRSKDD-dsRNA plus LPS and *Rlecs–*YRSKDD-dsRNA plus PGN groups compared with the *GFP*-dsRNA plus LPS, *GFP*-dsRNA plus PGN, LPS only and PGN only groups (figure 6*b*). The number of tested bacteria (*S. aureus* and *V. parahaemolyticus*) in the *Rlecs*–YRSKDD-dsRNA group was evidently higher than that in the control group at 10 and 20 min post-injection (figure 6*c*).

### 2.6. Sugar binding, bacteria binding, bacterial inhibition and bacterial clearance of Rlecs–YRSKDD with variable tandem repeats

**Figure 7.**
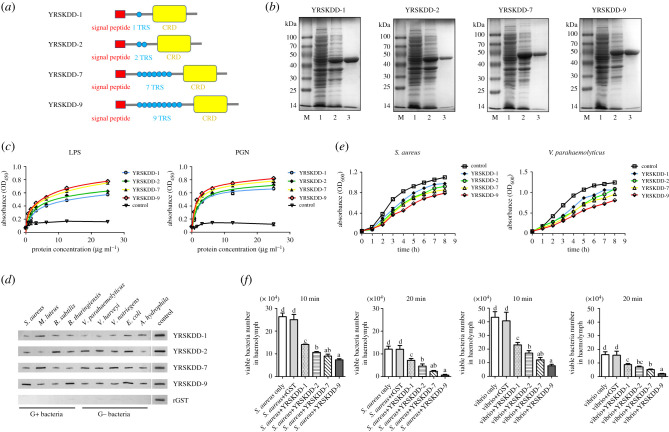
Analysis of sugar binding, bacteria binding, bacterial inhibition and bacterial clearance of rRlecs–YRSKDD with different numbers of tandem repeats. (*a*) Schematic of the domain composition of Rlec–YRSKDD-1, Rlec–YRSKDD-2, Rlec–YRSKDD-7 and Rlec–YRSKDD-9 (the numbers represent the number of tandem repeats). (*b*) SDS–PAGE of rRlec–YRSKDD-1, rRlec–YRSKDD-2, rRlec–YRSKDD-7 and rRlec–YRSKDD-9. Lane M: protein molecular standard; lane 1: total proteins of *E. coli* with recombinant plasmids without IPTG induction; lane 2: total proteins of *E. coli* with recombinant plasmids induced with 0.5 mM IPTG; lane 3: purified rRlec–YRSKDD-1, rRlec–YRSKDD-2, rRlec–YRSKDD-7 and rRlec–YRSKDD-9. (*c*) ELISA was used to detect the binding activities of rRlec–YRSKDD-1, rRlec–YRSKDD-2, rRlec-–YRSKDD-7, and rRlec–YRSKDD-9 to LPS and PGN. (*d*) Binding analysis of rRlec–YRSKDD-1, rRlec–YRSKDD-2, rRlec–YRSKDD-7 and rRlec–YRSKDD-9 to various Gram-positive and Gram-negative bacteria. (*e*) Bacterial growth inhibitory activities of rRlec–YRSKDD-1, rRlec–YRSKDD-2, rRlec–YRSKDD-7 and rRlec–YRSKDD-9. Antimicrobial activities against cultured *S. aureus* and *V. parahaemolyticus* were shown by the growth inhibition curves. (*f*) Bacterial clearance assay of rRlec–YRSKDD-1, rRlec–YRSKDD-2, rRlec–YRSKDD-7 and rRlec–YRSKDD-9 *in vivo*. Prawns were injected with *S. aureus* or *V. parahaemolyticus* preincubated with purified rRlecs–YRSKDD or GST. Bacterial number was detected at 10 and 20 min post-bacterial injection. Data were analysed using ANOVA and SPSS. Significant differences are marked with different letters (a–d).

*Rlecs*–YRSKDD with 1, 2, 7 and 9 tandem repeats (figure 7*a*) was successfully expressed in *Escherichia coli*, and the recombinant proteins with glutathione S-transferase (GST) tag were purified to study the effect of tandem repeat regions on the function of Rlecs (figure 7*b*). These four recombinant proteins with 1, 2, 7 and 9 tandem repeats were named rRlec–YRSKDD-1, rRlec–YRSKDD-2, rRlec–YRSKDD-7 and rRlec–YRSKDD-9, respectively. These recombinant proteins can bind directly to LPS and PGN in a concentration-dependent manner. However, their binding affinities towards LPS and PGN were different. rRlec–YRSKDD-9 had the highest binding activity, followed by rRlec–YRSKDD-7, rRlec–YRSKDD-2 and rRlec–YRSKDD-1 (figure 7*c*). rRlec–YRSKDD-1, rRlec–YRSKDD-2, rRlec–YRSKDD-7 and rRlec–YRSKDD-9 had wide binding activities towards diverse microorganisms (figure 7*d*). The bacterial inhibition results showed that rRlecs–YRSKDD had increased bacterial growth-inhibiting activity with increasing number of tandem repeats (figure 7*e*). The bacterial clearance rates in the rRlecs–YRSKDD groups were higher than that in the GST group at 10 and 20 min post-injection. The bacterial clearance rates of rRlecs–YRSKDD increased with the number of tandem repeats (figure 7*f*).

### 2.7. Effect of the number of tandem repeats in Rlecs–YRSKDD on the regulation of AMP gene expression and PO activity

**Figure 8.**
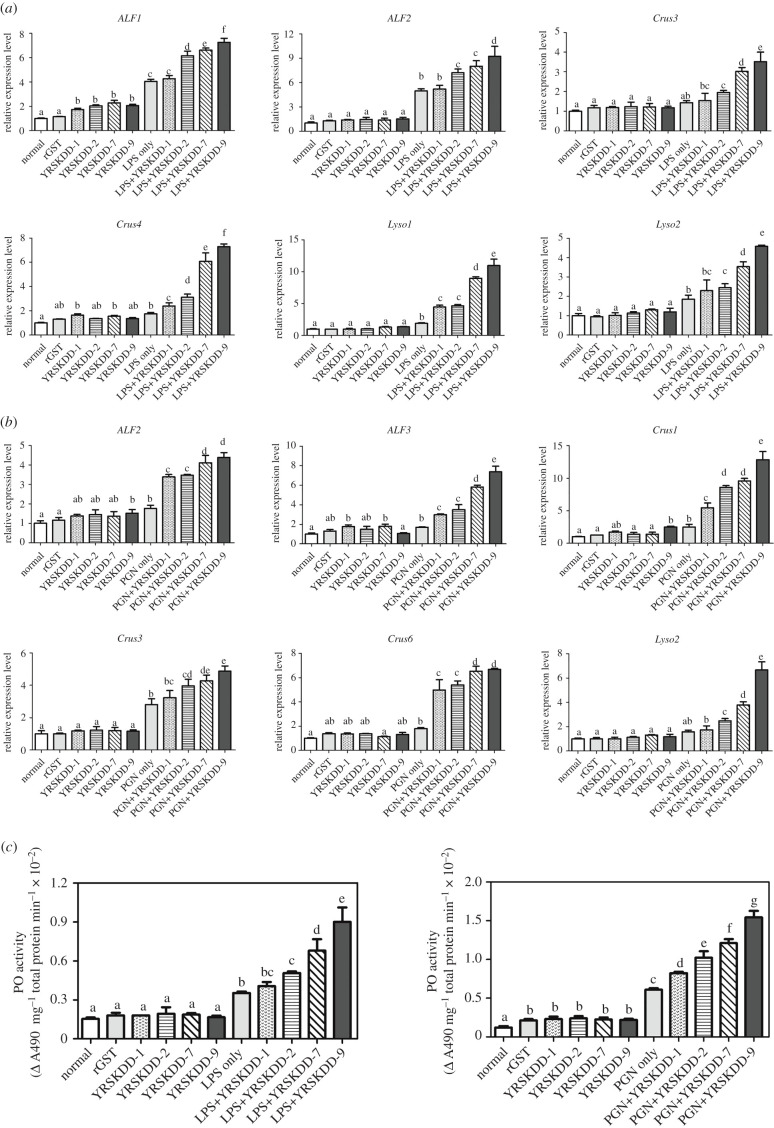
Analysis of *AMP* expression regulation and PO activation by rRlecs–YRSKDD with variable numbers of tandem repeats. Induction of *AMP* gene expression by rRlec–YRSKDD-1, rRlec–YRSKDD-2, rRlec–YRSKDD-7 and rRlec–YRSKDD-9. rRlecs–YRSKDD combined with LPS (*a*) or PGN (*b*) were injected into prawns. The expression level of *AMP* genes in gills were determined by qRT-PCR at 12 h post-injection. (*c*) Effect of rRlecs–YRSKDD on PO activity in the gills of prawns. Prawns were injected with rRlecs–YRSKDD plus LPS or PGN. The gills were collected at 12 h post-injection, and PO enzymatic activity was measured using l-DOPA as substrate and monitored by spectrophotometry at 490 nm. Each sample was composed of three prawns. Data were analysed by ANOVA. Results are expressed as mean ± s.d. derived from three independent experiments. Significant differences are marked with different letters (a–g).

LPS or PGN combined with purified rRlec***–***YRSKDD-1, rRlec***–***YRSKDD-2, rRlec***–***YRSKDD-7 or rRlec***–***YRSKDD-9 were injected to the prawns to study the effect of the number of tandem repeats in Rlecs***–***YRSKDD on the regulation of *AMP* gene expression and PO activity. The transcription levels of *ALF1*, *ALF2*, *Crus3*, *Crus4*, *Lyso1* and *Lyso2* were increased in rRlecs***–***YRSKDD plus LPS groups compared with the GST, LPS and rRlecs***–***YRSKDD only groups (figure 8*a*). Similarly, rRlec***–***YRSKDD-1, rRlec***–***YRSKDD-2, rRlec***–***YRSKDD-7 and rRlec***–***YRSKDD-9 with PGN groups had higher *AMP* induction activity compared with the control groups (figure 8*b*). The induction activity of *AMP* genes showed an overall upward trend with increased tandem repeat number in rRlecs***–***YRSKDD (figure 8*a,b*). The LPS plus rRlecs***–***YRSKDD and the PGN plus rRlecs***–***YRSKDD groups had higher PO activities compared with the control groups (figure 8*c*). rRlecs***–***YRSKDD with a higher number of tandem repeats showed higher PO induction activity than those with lower number of tandem repeats (figure 8*c*).

## 3. Discussion

Many different CTLs with diverse functions were reported in crustaceans. However, lectins belong to different families, and the number of their common features is small. In this study, a new lectin gene family (*Rlec*) with different numbers of repeat units, different arrangement patterns of repeat units, variable exon 5 sequences and hypervariability was found in prawns, and these isoforms originated from gene duplications. The changes in gene copy number provide a rapid mechanism for adaptation to new or different environments by using existing functional sequences to respond to changing conditions [18]. As this process repeats, gene duplication can lead to the expansion of gene families and provide important evolutionary fodder on which selection can facilitate adaptation [19]. In this study, each genomic sequence corresponded to a specific *Rlec* isoform transcript. Thus, a strong evolutionary pressure has driven the diversification of *Rlec* genes and gives rise to neo- or sub-functionalization and retention in the prawn genome. In addition, the tandem repeat regions of *Rlec* isoforms were composed of tandem arrangements of closely related motifs, which ranged from 1 to 9. Notably, the tandem repeats in the coding region were not identical and did not produce frameshift mutations, which were extremely rare, but the length of the repeat unit was 33 or 30 bp. In penaeid shrimp, dinucleotide repeats (AT)n, (AC)n and (AG)n accounted for 81.40% of the total simple sequence repeats, but most repeat sequences are located in the intergenomic regions (24.63%) and the introns of protein-coding genes (22.07%) [20]. The variation in tandem repeat copy number has been reported in humans [11], but the tandem repeats in the coding region are rare compared with those in the non-coding region because of the strong selection against repeats that result in frameshift mutations [21]. Several examples of slipped-strand mispairing can explain the characteristics of *Rlecs*' tandem repeat sequences in the coding region.

In addition to gene duplications, alternative splicing is another pathway that contributes to the diversity of *Rlecs* at the post-transcriptional level. Most eukaryotic protein-coding genes are interrupted by introns, which must be precisely removed from the precursor transcripts to produce functional mRNAs [22]. RNA splicing has been considered as the main driver of transcriptional diversity, and a large number of regulatory mechanisms have been described. Our results exhibited the three alternative splicing modes of *Rlec* genes. Different mRNA splicing isomers were produced in one mRNA precursor of *Rlecs* by various splicing methods (different splicing site combinations were selected). The selection of 5′ (3′) splice sites in *Rlec* genes can increase the diversity of *Rlec* genes. Despite the differences in sequence characteristics, most *Rlec* genes had similar structures, wherein six exons were interrupted by five introns, and some *Rlec* isoforms without exon 3 or 5 can be produced by exon skipping. Substantial gene expansion combined with alternative splicing can induce hypervariable *Rlec* isoforms, which may prompt the functional diversity or enhancement of the *Rlec* gene family in prawns.

The pathogen-associated molecular pattern–PRR interaction activates the immune cascade, which leads to changes in the expression of effector genes. The primary effector systems were the production of AMPs and PO-dependent melanization. AMP is a kind of peptide found in almost all living organisms. AMPs play very crucial roles in innate immunity by killing invading microorganisms and regulating other immune or inflammatory responses [23]. The prophenoloxidase (proPO) activation system is considered a vital innate defence mechanism in the immune system of crustaceans. Inactive proPO is transformed into active PO through a protease cascade reaction, after which the active PO catalyses the melanin biosynthesis pathway by hydroxylation. This catalysis leads to the oxidation of odiphenols to *o*-quinones and then non-specifically cross-links all molecules to form stable melanin, which directly kills and clears invading microbes [24]. Our results showed that LPS and PGN cannot upregulate the expression levels of *AMPs* and PO activity in *Rlecs*–YRSKDD (the main type of *Rlecs*) knockdown prawns. These results indicated that *Rlecs**–***YRSKDD played a vital role in the regulation of *AMP* expression and proPO system activation. Furthermore, the variation in the number of tandem repeats influenced the immune function of Rlecs***–***YRSKDD. The immune functions of rRlecs***–***YRSKDD, including sugar binding, microbial inhibition and clearance, regulation of *AMP* gene expression and PO activation, were enhanced as the number of tandem repeats increased. Tandemly repeated DNA sequences were the highly dynamic genome components, which can exert a regulatory influence on protein function. The variation in the number of tandem repeats in the genes provide the functional diversity of cell surface antigens that allow rapid adaptation to the environment and/or escape from the host immune system in fungi and other pathogens [3]. Studies on the evolution of such repeated sequences have focused on sequences found in the non-coding and the coding regions of the genome. A 40 bp variable number of tandem repeat (VNTR) polymorphism in the non-coding region of the dopamine transporter (*DAT*) gene does not alter the amino acid sequence of the protein. However, the function of DAT is altered, and VNTR is identified as a functional polymorphism [25]. In prokaryotes, the collagen-like protein (BclA) is the first identified *Bacillus anthracis* protein that contain an internal collagen-like region of GXX repeats, which is associated with phenotypic variation [26]. In vertebrates, VNTR polymorphism in the P-selectin glycoprotein ligand 1 gene has been associated with ischaemic cerebrovascular disease [27]. In humans, host defence proteins do not have an excess of tandem repeat variation [11]. However, such advantageous tandem repeat polymorphisms are predicted to occur in invertebrate host defence proteins [11]. In this study, we provided an example of VNTR polymorphism in the crustacean immune protein of Rlecs, and VNTR can alter the immune function of Rlecs.

The expansion of genes promotes functional diversification and affords organisms with great phenotypic flexibility [19]. Lectins are important PRRs that can recognize and bind bacteria. In general, invertebrates only rely on innate immunity to combat diverse pathogens, and innate immunity is generally considered non-specific. However, innate immune PRRs often have high specificity for target molecules. Adaptive immunity with specificity exists only in vertebrates [28]. However, the innate defence systems of invertebrates also exhibit some specific immune responses against specific pathogens [29,30]. In our study, the *Rlecs* in prawns underwent dramatic gene expansions. Different types of *Rlecs* varied in tissue distribution, expression level and response to bacterial challenge. Different types of *Rlecs* produced various responses towards a certain bacterium. Therefore, the expansion of the *Rlecs* gene family due to gene duplications may be used by prawns to recognize different pathogens.

## 4. Material and methods

### 4.1. Animals, tissue collection and RNA extraction

*Macrobrachium nipponense* (approx. 3–4 g) were obtained from an aquaculture market in Nanjing, Jiangsu Province, China. The prawns were cultured in tanks at 23–25°C with freshwater for one week before processing. LPS and PGN were purchased from Sigma (St Louis, MO, USA). *Staphylococcus aureus* (Luria–Bertani (LB) medium, 37°C), *Micrococcus luteus* (LB medium, 37°C), *Bacillus subtilis* (LB medium, 37°C), *Bacillus thuringiensis* (LB medium, 37°C), *V. parahaemolyticus* (LB medium, 28°C), *Vibrio harveyi* (tryptic soy broth (TSB) medium, 28°C), *Vibrio natriegens* (LB medium, 28°C), *E. coli* (LB medium, 37°C) and *Aeromonas hydrophila* (TSB medium, 28°C) were kept in our laboratory.

For the bacterial challenge experiments, the prawns were randomly divided into two groups, and each group contained 30 individuals. In two experimental groups, approximately 50 µl *S. aureus* (3 × 10^6^ cells) or *V. parahaemolyticus* (3 × 10^6^ cells) were injected into the abdominal segment of *M. nipponense* by using a 1 ml sterile syringe. The prawns in the control group were injected with the same volume of phosphate-buffered saline (PBS; 0.14 M NaCl, 3 mM KCl, 8 mM Na_2_HPO_4_ and 1.5 mM KH_2_PO_4_; pH 7.4). After treatment, the prawns were returned to the culture water tanks. Gills were randomly collected from five prawns from the experimental and control groups at 2, 6, 12 and 24 h post-injection. The heart, hepatopancreatic, gill, stomach and intestinal tissues and haemocytes were also collected from healthy prawns for RNA extraction. The haemolymph was extracted from the ventral sinus by using a 1 ml syringe containing one-third volume of anticoagulant buffer (acid citrate dextrose B (ACD-B): 1.47 g glucose, 0.48 g citric acid, 1.32 g trisodium citrate; made up to 100 ml volume by using double distilled water; pH 7.3) and then immediately centrifuged at 2000 r.p.m. for 10 min at 4°C for the isolation of hemocytes. Total RNA was extracted from the tissues by using the RNApure high-purity total RNA rapid extraction kit (Spin-column, BioTeke, Beijing, China) in accordance with the manufacturer's protocols. The RNA quality was assessed using electrophoresis on a 1.0% agarose gel. The first-strand cDNA of the samples for qRT-PCR was obtained through the PrimeScript 1st strand cDNA synthesis kit (Takara, Japan) with the Oligo(dT) primer. First-strand cDNA was synthesized using the 5′-CDS primer A and the SMARTer IIA oligo for 5′ fragment cloning and the 3′-CDS primer A for 3′ fragment cloning by using the SMARTer™ rapid amplification of CDNA ends (RACE) cDNA amplification kit (Clontech, Mountain View, CA, USA). All procedures were performed in accordance with the manufacturer's protocols.

### 4.2. Amplification of the intermediate sequences of 76 Rlecs isoforms and cloning of the full-length cDNA of *Rlec*–YRSKDD with nine tandem repeats

The partial sequence of a lectin gene with tandem repeats was found in the transcriptome database of *M. nipponense*. Two primers (Rlecs-RT-F and Rlecs-RT-R, table 1) were designed on the basis of the partial sequences to amplify the middle fragments by using gills, stomach and intestine cDNA as templates. More than one band can be amplified, and the mixed fragments were gel purified and cloned into the pEasy-T3 vector (TransGen Biotech, China). First, we selected 10 positive clones to sequence. Sequence analysis showed that these lectin genes had sequence diversity. Thus, more positive clones were selected for sequencing. Finally, 76 different *Rlec* genes were identified after removing the redundant sequences, and these genes were used to construct the phylogenetic tree.

**Table 1.** Sequences of the primers used in the study.

primers name	sequence (5′–3′)
**gene cloning** Rlecs-F	GCTTGCGACCATTTTTCTCCTCCTTGC
Rlecs-R	TTTCTGACGGCTCTGTTCCCAGGTGCT
Rlecs-RT-F	GCTTGCGACCATTTTTCTCCTCCTTGC
Rlecs-RT-R	ATCCAGTTGGGCAGGTTTCTCGCAGAT
**genome amplification** Rlecs-walk-F1	TTGCGACCATTTTTCTCCTCCTTGCAA
Rlecs-walk-F2	GCTGCCACTCGCAGCAATTGGATCATA
Rlecs-gF	TGAAGCTGAGATCCTCCTGTTACTTC
Rlecs-gR	ATCCAGTTGGGCAGGTTTCTCGCAGAT
Rlecs–CNDSGD-gF1	GCTTGCGACCATTTTTCTCCTCCTTGCAAT
Rlecs–CNDSGD-gR1 Rlecs–CNDSGD-gF2 Rlecs–CNDSGD-gR2 **qRT-PCR** Rlecs-qF Rlecs–FHYKGD-qR Rlecs–YRSKDD-qR Rlecs–FQSKDG-qR Rlecs–YHYQEH-qR Rlecs–YNYFDD-qR Rlecs–YTYKED-qR Rlecs–YYYKED-qR Rlecs–YVVSDD-qR β-actin-qF β-actin-qR **RNAi** Rlecs–YRSKDD-dsRNA-F Rlecs–YRSKDD-dsRNA-R GFP-dsRNA-F GFP-dsRNA-R Rlecs–YRSKDD-qF Rlecs–YRSKDD-qR ALF1-qF ALF1-qR ALF2-qF ALF2-qR ALF3-qF ALF3-qR Cru1-qF Cru1-qR Cru3-qF Cru3-qR Cru4-qF Cru4-qR Cru6-qF	ATCATTGAGGCGATCTCCGCTATCGTT TCGCCTCAATGATGTCCGTTGTTTCCAT ATCCAGTTGGGCAGGTTTCTCGCAGATAT AATCGTTGAATCATGGTGGG AGGCGATCGCCTTTGTAGTG AGGCGATCGTCTTTGGATCG AAGCGACCGTCTTTGGATTG AGGCGATGTTCTTGGTAATG AGGCGATCGTCAAAGTAGTT ATGCGATCTTCTTTGTAGGT AAGCGATCTTCTTTGTAGTA AAGCGATCGTCGCTGACAAC TATGCACTTCCTCATGCCATC AGGAGGCGGCAGTGGTCAT GCGTAATACGACTCACTATAGGATGTTCAACTTATTGGCCATAG GCGTAATACGACTCACTATAGGAATCCAGTTGGGCAGGTTTCTC GCGTAATACGACTCACTATAGGTGGTCCCAATTCTCGTGGAAC GCGTAATACGACTCACTATAGGCTTGAAGTTGACCTTGATGCC AATCGTTGAATCATGGTGGG AGGCGATCGTCTTTGGATCG GTGGTGCCCAGGATGGACTT AGAGGATGGTGGAGGAAATT AGAACCACCTGAACCCAACG TGACAGATTAAGCCAGCCCC GTCGATGGAGTGTATGATGAGG GTAGTGCAGCTCGAGTCTTT TTTGGTTTCTGGCATTTCC CTTGTTGCTGTCACCGCTC CCTGACACCATTCCCTTCG GCCGCCACCAGACCCAACT GGAATTAGAAGGGCCCGTCGG TCATAGCAGCACTTGTCAGCG CTCCGTGTCCTCCCATACC
Cru6-qR Lyso1-qF Lyso1-qR Lyso2-qF Lyso2-qR **recombinant expression** Rlecs–YRSKDD-6P-2-ex-F Rlecs–YRSKDD-6P-2-ex-R	AGTTCCCTGTCGACTTCCT GGCAGTGGGAGAAGAAGAAA TGAACCAGGACGAAGATGATG TTAGTGGTTTCAGCGGTCTC CGGGACATCTTGTGTTTGTTC GGATCCCCAGGAATTCCCCAGCAATTGGATAATATTCAGC GATGCGGCCGCTCGAGTTAATCCAGTTGGGCAGGTTTCTC

The obtained *Rlec* genes showed some differences, but their sequences had high similarity. Thus, designing primers to amplify the full length of a unique lectin gene was difficult. Rlecs-F and Rlecs-R were designed in accordance with the conservative region of the 76 *Rlec* genes. 5′ and 3′ RACE were performed using Rlecs-R and Rlecs-F, respectively (table 1). 5′- and 3′-RACE-ready cDNA were synthesized using the Clontech SMARTer™ RACE cDNA amplification kit (Takara). The PCR volume was 50 µl (2.5 µl of 5′-RACE-ready cDNA or 3′-RACE-ready cDNA, 5 µl of 10 × Advantage 2 PCR buffer, 1 µl of 10 mM dNTPs, 1 µl of 10 mM Rlecs-R or Rlecs-F, 5 µl of Universal Primer A mix, 34.5 µl of PCR-grade water and 1 µl of 50× Advantage 2 polymerase mix). The PCR conditions were set as follows: 5 cycles of 94°C for 30 s and 72°C for 3 min, 5 cycles of 94°C for 30 s and 70°C for 30 s, and 25 cycles of 94°C for 30 s, 68°C for 30 s and 72°C for 3 min. The 5′- and the 3′-RACE fragments were then cloned into the pEasy-T3 vector (TransGen Biotech, People's Republic of China). A number of positive clones were selected for sequencing. Numerous 5′ and 3′ cDNA sequences were obtained because this lectin gene family had gene diversity. Only the overlaps of the 5′- and the 3′-end sequences were identical and can be spliced into a gene. We successfully obtained the full-length cDNA of *Rlec*–YRSKDD with nine tandem repeats.

### 4.3. Bioinformatics analysis of Rlec gene family

The gene translation and prediction of the deduced protein were conducted using the ExPASy (https://web.expasy.org/translate/). Multiple sequence alignments were generated using the ClustalW2 program (http://www.ebi.ac.uk/tools/clustalw2). The MEGA 7.0 was used to produce phylogenetic trees, and the neighbour-joining method was used for phylogenetic analysis [31].

### 4.4. Amplification of *Rlec* DNA sequences

A total of 76 *Rlec* genes that belonged to a lectin gene family were identified. Whether each *Rlec* isoform had a corresponding DNA sequence in the genome was not known. Therefore, the genome sequences of *Rlecs* needed to be amplified. Direct amplification by using genome DNA as template and genome walking were used to obtain the DNA of *Rlecs*. Genome walking was performed using the primers (Rlecs-walk-F1 and Rlecs-walk-F2) in table 1. The Universal GenomeWalker kit (Clontech, USA) was used to amplify the genomic sequences of *Rlecs*. Experiments were performed in accordance with the protocols of the kit. The *Rlecs* DNA with variable numbers of tandem repeats were obtained after analysing the sequencing results, which indicated that each *Rlec* transcript had its corresponding DNA sequence. Two primers (Rlecs-gF and Rlecs-gR) were designed to amplify the *Rlecs* DNA sequences directly. Multiple *Rlecs* DNA sequences corresponding to different types of *Rlecs* were identified. This finding also confirmed that each *Rlec* transcript had a corresponding DNA sequence. However, two sets of amplified *Rlec* DNA sequences were not matched because of the diversity of the *Rlec* genes. The results of genome walking and direct PCR amplification showed that the *Rlecs* DNA had six exons interrupted by five introns. Next, we selected *Rlecs*–CNDSGD to amplify its DNA sequence. Two pairs of primers (Rlecs–CNDSGD-gF1, Rlecs–CNDSGD-gR1; Rlecs–CNDSGD-gF2, Rlecs–CNDSGD-gR2) were designed for the direct PCR amplification of the *Rlecs*–CNDSGD DNA sequence. Among these two pairs of primers, Rlecs–CNDSGD-gR1 and Rlecs–CNDSGD-gF2 were specific to *Rlecs*–CNDSGD. Finally, the complete genome sequence of *Rlec*–CNDSGD-8 can be obtained by splicing the two amplified fragments.

### 4.5. Tissue distribution and expression pattern analysis of the eight types of *Rlecs* upon bacterial challenge

Fifteen types of *Rlec* genes were identified in this study, but designing primers for fluorescence quantification to distinguish all types of *Rlec* genes was difficult. Thus, we selected eight types of *Rlec* genes to study their tissue distribution and expression change upon bacterial challenge. The position of primer binding is shown in figure 1*b*. The forward primer (Rlecs-qF) used was the same, whereas the reverse primer was specific to the unique type of *Rlecs*. qRT-PCR was used to study the distribution of these genes in the tissues of the haemocytes, hearts, hepatopancreases, gills, stomachs and intestines of healthy prawns. The expression patterns of the eight types of *Rlec* genes in the gills of *M. nipponense* challenged by *S. aureus* or *V. parahaemolyticus* at 2, 6, 12 and 24 h were also determined by qRT-PCR. qRT-PCR was carried out using 2× SYBR Premix Ex Taq kit (Takara, People's Republic of China) and the following primers: Rlecs-qF and Rlecs–FHYKGD-qR/Rlecs–YRSKDD-qR/Rlecs–FQSKDG-qR/Rlecs–YHYQEH-qR/ Rlecs–YNYFDD-qR/Rlecs–YTYKED-qR/Rlecs–YYYKED-qR/Rlecs–YVVSDD-qR (table 1). qRT-PCR was set under the following conditions: 95°C for 30 s, followed by 40 cycles of 95°C for 5 s, 60°C for 20 s and a melting curve analysis from 65°C to 95°C. β-Actin was used as the reference internal control. The relative mRNA expression was calculated using the 2^−ΔΔCt^ method [32]. PCR amplification was performed in triplicate. Unpaired sample *t*-test and one-way analysis of variance (ANOVA) were performed for statistical analysis, and the difference was considered significant if *p* < 0.05.

### 4.6. *In vivo* RNA interference of *Rlecs*–YRSKDD and detection of AMP expression and PO activity

*Rlecs*–YRSKDD is the main type of *Rlecs*. Thus, we selected *Rlecs*–YRSKDD for the RNA interference study. A pair of primers (Rlecs–YRSKDD-dsRNA-F, Rlecs–YRSKDD-dsRNA-R) with T7 promoter at the 5′ end of the primer was designed to amplify the DNA templates of *Rlecs*–YRSKDD. *GFP* was also amplified to synthesize the *GFP*-dsRNA as control by using the primers of GFP-dsRNA-F and GFP-dsRNA-R. *Rlecs*–YRSKDD-dsRNA and *GFP*-dsRNA were synthesized using an *in vitro* transcription T7 kit (Fermentas, USA). *Rlecs*–YRSKDD-dsRNA or *GFP*-dsRNA (30 µg) was injected into the second abdominal segment of prawns in the RNAi experiment. LPS or PGN (50 µl, 80 µg ml^−1^) was injected into the same prawns at 36 h post-injection. LPS or PGN (4 µg) alone were also injected into the prawns of the control groups. The gills of five prawns from each group were collected after 12 h for the detection of RNAi efficiency by qRT-PCR with the primers Rlecs–YRSKDD-qF and Rlecs–YRSKDD-qR (table 1).

The mRNA expression levels of the nine *AMP* genes, including *ALF1* (ALF1-qF and ALF1-qR), *ALF2* (ALF2-qF and ALF2-qR), *ALF3* (ALF3-qF and ALF3-qR), *Crus1* (Cru1-qF and Cru1-qR), *Crus3* (Cru3-qF and Cru3-qR), *Crus4* (Cru4-qF and Cru4-qR), *Crus6* (Cru6-qF and Cru6-qR), *Lyso1* (Lyso1-qF and Lyso1-qR) and *Lyso2* (Lyso2-qF and Lyso2-qR), were also analysed. All primer sequences used are shown in table 1. l-3,4-dihydroxy-phenylalanine (l-DOPA) was used as substrate for PO activity detection by following existing methods [33]. PO activity was monitored using spectrophotometry at 490 nm and defined as ΔA490 mg total protein^−1^ min^−1^. Five prawns from each experimental group (normal, LPS or PGN only, *GFP*-dsRNA + LPS or *GFP*-dsRNA + PGN, and *Rlecs*–YRSKDD-dsRNA + LPS or *Rlecs*–YRSKDD-dsRNA + PGN) were selected for PO activity detection. Results were analysed by ANOVA in the Statistical Package for Social Sciences (SPSS) v. 16.0, and *p* < 0.05 was considered significant.

### 4.7. Recombinant expression and purification of Rlecs–YRSKDD with 1, 2, 7 and 9 tandem repeats

A pair of primers, namely Rlecs–YRSKDD-6P-2-ex-F and Rlecs–YRSKDD-6P-2-ex-R (table 1), was designed to amplify *Rlecs*–YRSKDD. The primers were bound to the conserved region of *Rlecs*–YRSKDD. Thus, the *Rlecs*–YRSKDD with different numbers of tandem repeats were amplified. These amplified *Rlecs*–YRSKDD mixture were then inserted into pGEX-6P-2 vectors with digestion by *EcoR* І and *Xho* І (NEB, USA). Then, recombinant plasmids with *Rlecs*–YRSKDD were selected by sequencing. Finally, we selected the recombinant plasmids with *Rlec*–YRSKDD-1, *Rlec*–YRSKDD-2, *Rlec*–YRSKDD-7 and *Rlec*–YRSKDD-9 (the numbers represent the number of tandem repeats) for further transformation into *E. coli* BL21 (DE3) cells for expression. rRlecs–YRSKDD proteins with N-terminal GST tag were purified using glutathione Sepharose 4B chromatography (Gen-Script, USA) in accordance with the manufacturers' protocols. Purified protein was separated using 12% reducing sodium dodecyl sulfate (SDS)–polyacrylamide gel electrophoresis (PAGE) and visualized using Coomassie brilliant blue R250.

### 4.8. Sugar and microbial binding assays of rRlecs–YRSKDD

rRlec–YRSKDD-1, rRlec–YRSKDD-2, rRlec–YRSKDD-7 and rRlec–YRSKDD-9 were used for sugar and microbial binding assays and in the following function experiments to study the effect of tandem repeats on the function of Rlecs. The ability of rRlecs–YRSKDD to bind directly to LPS and PGN was analysed through enzyme-linked immunosorbent assay. LPS or PGN (50 µl, 80 µg ml^−1^) were coated on a 96-well microlitre plate, incubated overnight at 37°C, and heated at 60°C for 30 min. Non-specific adsorption was prevented by blocking each well with 200 µl of 1 mg ml^−1^ bovine serum albumin (BSA)–Tris-buffered saline (TBS) at 37°C for 2 h, and the wells were washed with 200 µl TBS four times. Subsequently, purified rRlec–YRSKDD-1, rRlec–YRSKDD-2, rRlec–YRSKDD-7 or rRlec–YRSKDD-9 with different concentrations (0.78, 1.56, 3.125, 6.25, 12.5 and 25 µg ml^−1^) in BSA–TBS (0.1 mg ml^−1^) were added to the wells and incubated at room temperature for 3 h. The same concentration of GST protein was used as control. The plates were washed with TBS (200 µl well^−1^) four times and then incubated with 100 µl of rabbit monoclonal anti-GST antibody (1 : 2000 dilution in 0.1 mg ml^−1^ BSA–TBS), which was the primary antibody, at 37°C for 2 h. The plate was washed as above and then incubated with peroxidase-conjugated goat anti-rabbit IgG (1 : 5000 dilution in 0.1 mg ml^−1^ BSA–TBS), which was the secondary antibody, at 37°C for 1 h. The colour was developed with 100 µl of 0.01% 3,3′,5,5′-tetramethylbenzidine (Sigma), and the reaction was stopped using 2 M H_2_SO_4_. Absorbance was read at 450 nm with a plate reader (BioTek Instruments, USA). Three biological repeats were used for each group. The results were expressed as mean ± s.d. and analysed using ANOVA and SPSS.

Nine species, including Gram-positive (*S. aureus*, *M. luteus*, *B. subtilis* and *B. thuringiensis*) and Gram-negative (*V. parahaemolyticus*, *V. harveyi*, *V. natriegens*, *E. coli*, and *A. hydrophila*) bacteria, were used for the assay. Approximately 500 µl of rRlec–YRSKDD-1, rRlec–YRSKDD-2, rRlec–YRSKDD-7 or rRlec–YRSKDD-9 was incubated with microbes (2.0 × 10^8^ cells ml^−1^) in the midlogarithmic phase by gentle rotation for 30 min at room temperature. After centrifugation, the harvested cells were washed thrice with TBS, eluted with 5% SDS and boiled for 1 min. The binding between microbes and recombinant protein was analysed through 12.5% SDS–PAGE and detected using western blot. An anti-GST antibody (TransGen, China) was used. The bacterial cells used as controls were incubated with the GST protein and subjected to the same treatments. The experiments were repeated thrice.

### 4.9. Microbial inhibition and clearance assay of rRlecs–YRSKDD

The growth curves of *S. aureus* and *V. parahaemolyticus* cultured with rRlec–YRSKDD-1, rRlec–YRSKDD-2, rRlec–YRSKDD-7 or rRlec–YRSKDD-9 were tested as previously reported with some modification [34]. In brief, *S. aureus* or *V. parahaemolyticus* in logarithmic phase were centrifuged, washed with TBS and resuspended in TBS (2.0 × 10^8^ cells ml^−1^). Approximately 50 µl of *S. aureus* or *V. parahaemolyticus* was transferred into 5 ml LB broth, and rRlec–YRSKDD-1, rRlec–YRSKDD-2, rRlec–YRSKDD-7 or rRlec–YRSKDD-9 was added to a final concentration of 100 µg ml^−1^. TBS and GST were used as blank and negative controls, respectively. Each sample was incubated at 37°C with shaking at 200 r.p.m. The optical density at 600 nm was measured every 1 h from 0 to 8 h to detect the growth of *S. aureus* and *V. parahaemolyticus*. Each treatment was repeated thrice.

For the bacterial clearance assay of Rlecs–YRSKDD, purified rRlec–YRSKDD-1, rRlec–YRSKDD-2, rRlec–YRSKDD-7 or rRlec–YRSKDD-9 (600 µg ml^−1^, 500 µl) was incubated with 500 µl of *S. aureus* or *V. parahaemolyticus* in PBS (2 × 10^8^ cells) at room temperature for 30 min with gentle rotation. The same microbial counts for *S. aureus* or *V. parahaemolyticus* were incubated with GST (600 µg ml^−1^, 500 µl) or PBS (500 µl) as controls. After incubation, 50 µl of the mixture was injected into the prawns. The haemolymph of five prawns were extracted at 10 and 20 min post-injection by mixing with an equal volume of ACD-B anticoagulant. The samples were diluted 1000 times, and 50 µl of diluted sample was plated onto LB broth plates. The plates were incubated overnight at 37°C, and the number of bacterial colonies was counted. Each experiment was repeated thrice. Results were expressed as mean ± s.d., and data were analysed using ANOVA.

*Rlecs*–YRSKDD were knocked down to further study the bacterial clearance activity of *Rlecs*–YRSKDD. Approximately 30 µg of dsRNA (*Rlecs*–YRSKDD-dsRNA or *GFP*-dsRNA) was injected into the prawns. After 36 h, *S. aureus* or *V. parahaemolyticus* was injected into the normal and dsRNA-injected prawns. Then, the bacterial clearance assay was performed in accordance with the methods mentioned above.

### 4.10. The effect of tandem repeat numbers in rRlecs–YRSKDD on AMP expression and PO activity

The effects of the tandem repeat number in rRlecs–YRSKDD on *AMP* gene expression and PO activity were studied. First, rRlec–YRSKDD-1, rRlec–YRSKDD-2, rRlec–YRSKDD-7 or rRlec–YRSKDD-9 (2 µg) with LPS or PGN (4 µg) were injected into the prawns with a microsyringe. The injection of GST, LPS, PGN, rRlec–YRSKDD-1, rRlec–YRSKDD-2, rRlec–YRSKDD-7 or rRlec–YRSKDD-9 served as controls. The gills of five prawns in each group were sampled at 12 h post-injection for RNA or protein extraction. Nine *AMP* genes (*ALF1*, *ALF2*, *ALF3*, *Crus1*, *Crus3*, *Crus4*, *Crus6*, *Lyso1* and *Lyso2*) were analysed by qRT-PCR. The protein samples collected were used for the PO activity assay in accordance with the methods mentioned above. Results were analysed by ANOVA, and significant differences were accepted at *p* < 0.05.

## Supplementary Material

Electronic supplementary material

## Supplementary Material

Figure S1

## Data accessibility

All data are included in the manuscript figures and electronic supplementary material. All sequences in the manuscript have been submitted to GenBank. For the serial numbers, refer to electronic supplementary material, table S1.
