# Supplementary material for: Immune activation by a multigene family of lectins with variable tandem repeats in oriental river prawn (Macrobrachium nipponense)
Source: Open Biol. 2020 Sep 16;10(9):200141. doi: 10.1098/rsob.200141 (PMC7536079; doi:10.1098/rsob.200141)
Supplement: Electronic supplementary material [file rsob200141supp1.docx]

**Immune activation by a multigene family of lectins with variable tandem repeats in oriental river prawn (*Macrobrachium nipponense*)**

**Ying Huang ^1,2^, Xin Huang ^1^, Xuming Zhou ^5^, Jialin Wang ^4^, Ruidong Zhang ^1^, Futong Ma ^1^, Kaiqiang Wang ^1^, Zhuoxing Zhang ^1^, Xiaoling Dai ^1^, Xueying Cao ^1^, Chao Zhang ^1^, Keke Han ^1^ and Qian Ren ^1,3*^**

^1^ College of Marine Science and Engineering, Nanjing Normal University, 1 Wenyuan Road, Nanjing, Jiangsu, 210023, China

^2^ College of Oceanography, Hohai University, 1 Xikang Road, Nanjing, Jiangsu, 210098, China

^3^ Co-Innovation Center for Marine Bio-Industry Technology of Jiangsu Province, Lianyungang, Jiangsu, 222005, China

^4^ Hubei Key Laboratory of Genetic Regulation and Integrative Biology, School of Life Sciences, Central China Normal University, Wuhan, 430079, China

^5^ Key Laboratory of Animal Ecology and Conservation Biology, Institute of Zoology, Chinese Academy of Sciences, Beijing, China

*To whom corresponding should be addressed: Dr. Qian Ren, College of Marine Science and Engineering, Nanjing Normal University, 1 Wenyuan Road, Nanjing, Jiangsu, 210023, China. E-mail address: [renqian0402@126.com](mailto:renqian0402@126.com)

Ying Huang, Xin Huang and Xuming Zhou are equally contributed to this paper.

**Running title: hypervariable lectin genes in prawn**

**OPEN BIOLOGY. DOI: 10.1098/rsob.20160049**

**Supplementary Table S1. Characteristics of tandem repeats in 76 Rlecs.**

| **Name** | **The number of tandem repeats** | **Tandem repeat patterns** | **Types of Rlecs** | **GenBank accession numbers** |
| --- | --- | --- | --- | --- |
| 1-1 | 1 | 33 | YVVSDD | MN867280 |
| 1-2 | 1 | 33 | YYYKED | MN867281 |
| 1-3 | 1 | 33 | YRSKDD | MN867282 |
| 1-4 | 1 | 33 | FHYKGD | MN867283 |
| 1-5 | 1 | 33 | FHFKGD | MN867284 |
| 2-1 | 2 | 33-30 | FQSKDG | MN867285 |
| 2-2 | 2 | 33-30 | YNYFDD | MN867286 |
| 2-3 | 2 | 33-30 | YKKKED | MN867287 |
| 2-4 | 2 | 33-33 | YRSKDD | MN867288 |
| 2-5 | 2 | 33-33 | YTYKKD | MN867289 |
| 2-6 | 2 | 33-33 | YTYKED | MN867290 |
| 2-7 | 2 | 33-30 | FHYKGD | MN867291 |
| 2-8 | 2 | 33-33 | YRSKDD | MN867292 |
| 2-9 | 2 | 33-33 | YTYKED | MN867293 |
| 2-10 | 2 | 33-30 | YRSKDD | MN867294 |
| 2-11 | 2 | 33-33 | FQSKDG | MN867295 |
| 2-12 | 2 | 33-30 | YNYFDD | MN867296 |
| 2-13 | 2 | 33-33 | YRSKDD | MN867297 |
| 2-14 | 2 | 33-30 | YKKKED | MN867298 |
| 2-15 | 2 | 33-30 | YNYFDD | MN867299 |
| 2-16 | 2 | 33-30 | YNYFDD | MN867300 |
| 2-17 | 2 | 33-30 | YVVSDD | MN867301 |
| 2-18 | 2 | 33-30 | FQSKDG | MN867302 |
| 2-19 | 2 | 33-30 | YTYKED | MN867303 |
| 3-1 | 3 | 33-30-30 | YNYFDD | MN867304 |
| 3-2 | 3 | 33-30-30 | YHSKDD | MN867305 |
| 3-3 | 3 | 33-30-30 | CNDSGD | MN867306 |
| 3-4 | 3 | 33-30-33 | FHYKGD | MN867307 |
| 3-5 | 3 | 33-33-33 | FHYKGD | MN867308 |
| 3-6 | 3 | 33-30-30 | YRSKDD | MN867309 |
| 3-7 | 3 | 33-30-30 | YVVSDD | MN867310 |
| 3-8 | 3 | 33-30-33 | YVVSDD | MN867311 |
| 3-9 | 3 | 33-30-33 | FHFKGD | MN867312 |
| 3-10 | 3 | 33-33-33 | FHFKGD | MN867313 |
| 4-1 | 4 | 33-30-30-33 | FHYKGD | MN867314 |
| 4-2 | 4 | 33-30-30-33 | YYYKED | MN867315 |
| 4-3 | 4 | 33-30-33-33 | YKKRED | MN867316 |
| 4-4 | 4 | 33-30-30-33 | FHYKGD | MN867317 |
| 4-5 | 4 | 33-33-30-33 | FQSKDG | MN867318 |
| 4-6 | 4 | 33-30-33-33 | FQSKDG | MN867319 |
| 4-7 | 4 | 33-30-33-33 | FHYKGD | MN867320 |
| 4-8 | 4 | 33-30-33-33 | YRSKDD | MN867321 |
| 4-9 | 4 | 33-30-30-33 | YNYFDD | MN867322 |
| 4-10 | 4 | 33-30-30-33 | YRSKDD | MN867323 |
| 4-11 | 4 | 33-30-30-33 | FHFKGD | MN867324 |
| 5-1 | 5 | 33-30-33-30-33 | YYYKED | MN867325 |
| 5-2 | 5 | 33-30-33-30-33 | YNYFDD | MN867326 |
| 5-3 | 5 | 33-30-33-30-33 | YRSKDD | MN867327 |
| 5-4 | 5 | 33-30-33-30-33 | YTYKED | MN867328 |
| 5-5 | 5 | 33-33-30-30-33 | YYYKED | MN867329 |
| 5-6 | 5 | 33-33-33-30-33 | FHYKGD | MN867330 |
| 6-1 | 6 | 33-30-33-30-30-33 | FHYKGD | MN867331 |
| 6-2 | 6 | 33-30-30-33-30-33 | FHFKGD | MN867332 |
| 6-3 | 6 | 33-30-30-33-30-33 | YRSKDD | MN867333 |
| 6-4 | 6 | 33-30-30-30-33-33 | YTYKED | MN867334 |
| 6-5 | 6 | 33-33-30-30-33-33 | YRSKDD | MN867335 |
| 6-6 | 6 | 33-33-30-30-33-33 | YTYKED | MN867336 |
| 6-7 | 6 | 33-30-30-33-30-33 | YYYKED | MN867337 |
| 6-8 | 6 | 33-30-33-33-33-33 | FHFKGD | MN867338 |
| 6-9 | 6 | 33-30-30-33-30-33 | FHFKGG | MN867339 |
| 6-10 | 6 | 33-30-30-33-30-33 | FQSKDG | MN867340 |
| 7-1 | 7 | 33-33-33-33-33-33-33 | FHYKGD | MN867341 |
| 7-2 | 7 | 33-33-33-33-33-33-33 | YHYQEH | MN867342 |
| 7-3 | 7 | 33-30-33-33-33-33-33 | FHFKGD | MN867343 |
| 7-4 | 7 | 33-30-30-30-33-30-33 | YRSKDD | MN867344 |
| 7-5 | 7 | 33-30-30-30-30-30-33 | FQSKDG | MN867345 |
| 7-6 | 7 | 33-33-33-33-33-33-33 | FQSKDG | MN867346 |
| 8-1 | 8 | 33-30-30-30-30-30-30-30 | CNDSGD | MN867347 |
| 8-2 | 8 | 33-30-30-30-30-33-30-33 | YRSKDD | MN867348 |
| 8-3 | 8 | 33-30-33-33-33-33-33-33 | FHFKGD | MN867349 |
| 8-4 | 8 | 33-30-33-33-33-33-33-33 | FHYKGD | MN867350 |
| 8-5 | 8 | 33-30-33-33-33-33-33-33 | FHFKGD | MN867351 |
| 8-6 | 8 | 33-30-33-33-33-33-33-33 | FHFKGD | MN867352 |
| 8-7 | 8 | 33-33-33-33-33-33-33-33 | FQSKDG | MN867353 |
| 9-1 | 9 | 33-30-30-30-30-30-33-30-33 | YRSKDD | MN867354 |
| 9-2 | 9 | 33-30-30-30-30-30-30-33-33 | FHYKGD | MN867355 |


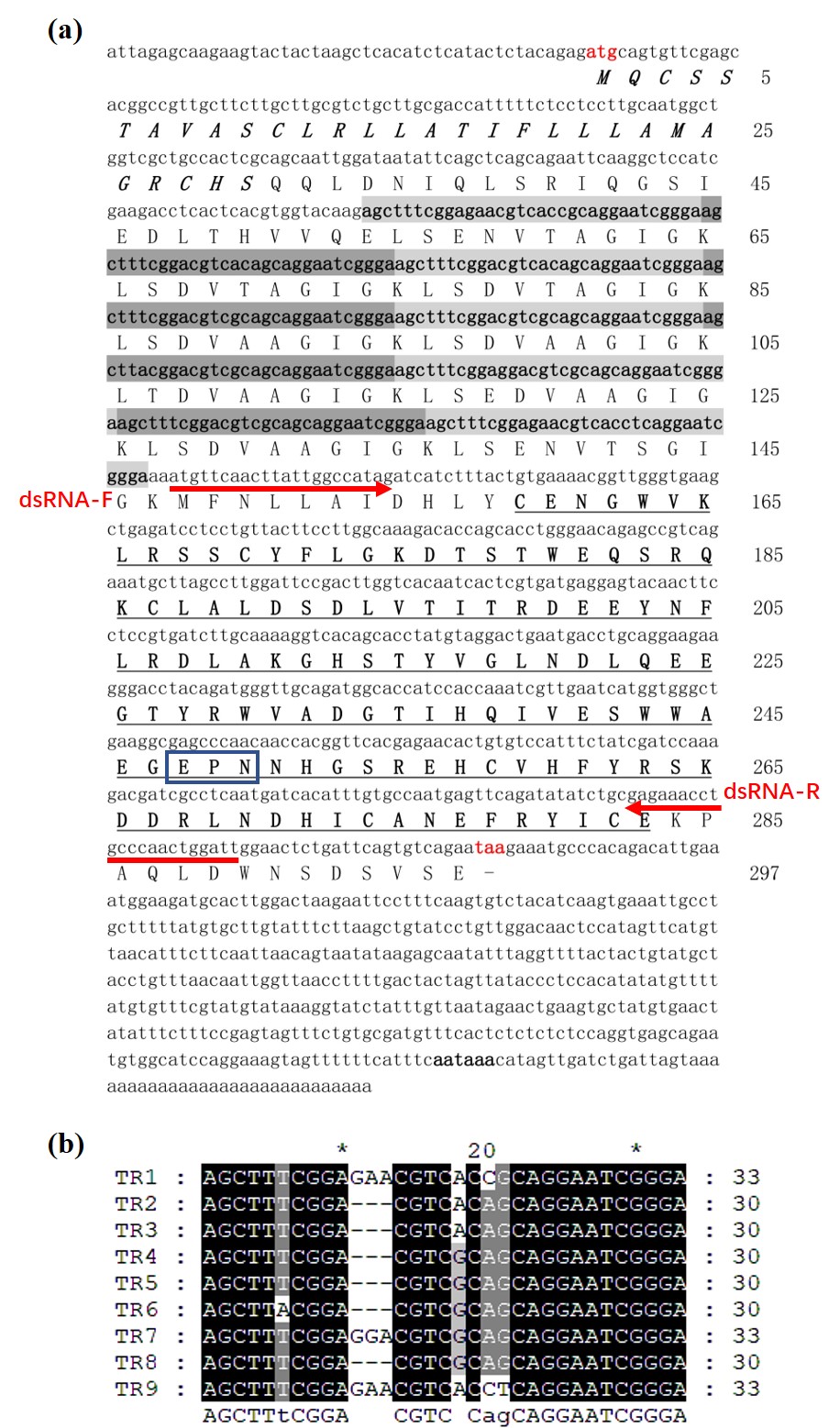


**Supplementary Figure S1. Sequence analysis of *Rlec*–YRSKDD containing nine tandem repeats.** (**a**) Nucleotides and deduced amino acid sequences of *Rlec*–YRSKDD-9 from *M. nipponense*. Nine tandem repeats in the nucleotide sequences of *Rlec* are indicated by light and dark gray shading. Start (ATG) and stop codons (TAA) are shown in red. The 3ʹ UTR containing the polyadenylation signal (AATAAA) is shown in bold. The signal peptide is shown in italics, and the conserved CRD is marked by a single underline. EPN is represented by a box. The primers of RNAi (Rlecs–YRSKDD-dsRNA-F and Rlecs–YRSKDD-dsRNA-R) are indicated by red arrows. (**b**) Multiple alignments of the nine tandem repeat units of *Rlec*–YRSKDD by using the ClustalW. Absolutely consistent sites and highly consistent sites are colored in dark and gray backgrounds, respectively.
