# Supplementary figures and images for: Immune activation by a multigene family of lectins with variable tandem repeats in oriental river prawn (Macrobrachium nipponense)
Source: Open Biol. 2020 Sep 16;10(9):200141. doi: 10.1098/rsob.200141 (PMC7536079; doi:10.1098/rsob.200141)

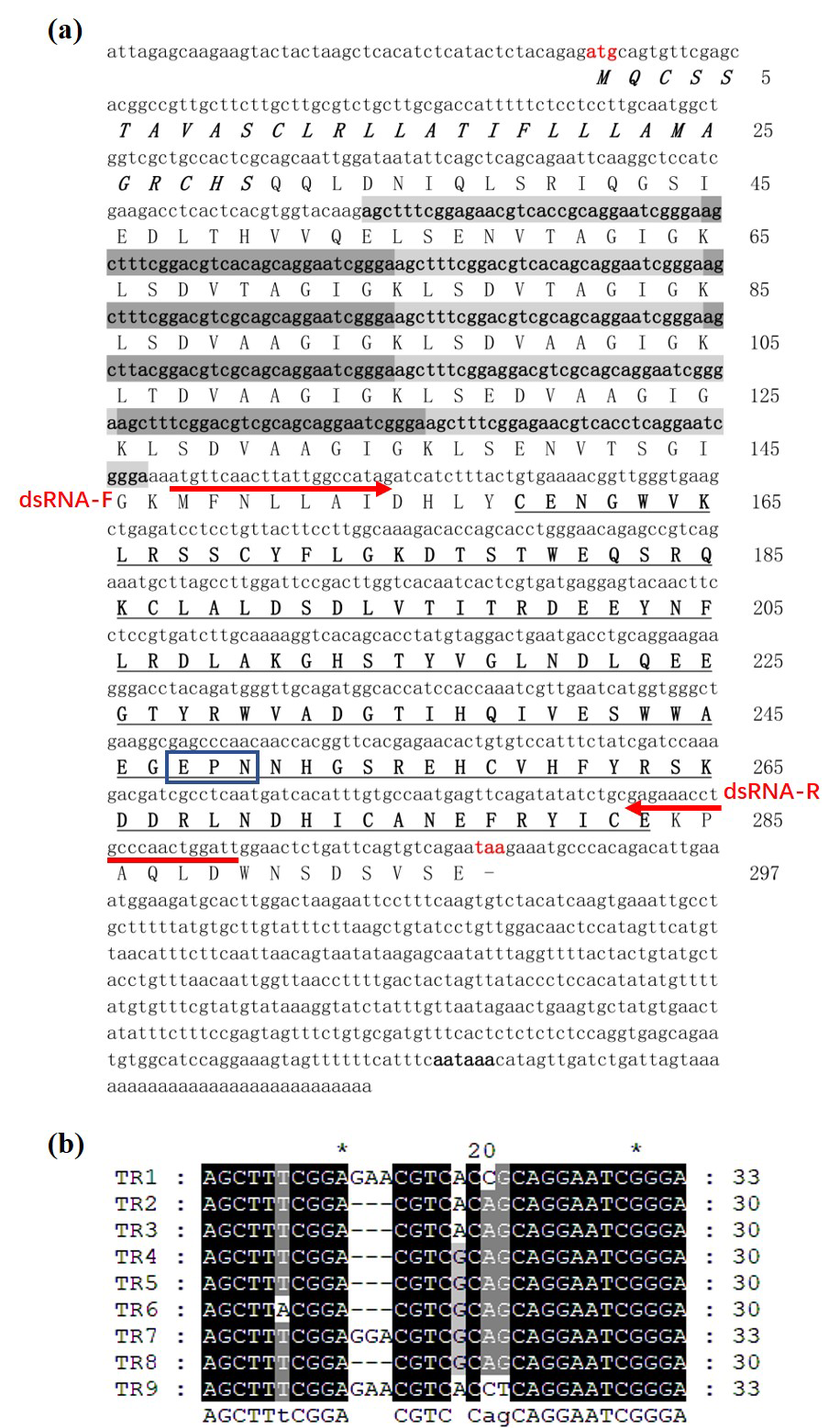

Supplement: Figure S1 [file rsob200141supp2.tif]
